# Supplementary material for: Association between objectively measured walking steps and sleep in community-dwelling older adults: A prospective cohort study
Source: PLoS One. 2020 Dec 14;15(12):e0243910. doi: 10.1371/journal.pone.0243910 (PMC7735606; doi:10.1371/journal.pone.0243910)
Supplement: Attachment — Submitted filename: Response to Reviewers.docx [file pone.0243910.s001.docx]

We would like to take this opportunity to express our sincere thanks to the reviewer who identified areas of the manuscript that needed corrections or modification. Based on the instructions provided in the decision letter and comments provided by the reviewers, we have revised the manuscript by modifying the relevant sections in the manuscript. Also appended below are point-by-point responses to the comments raised by the reviewers. We hope that our revisions along with our responses address reviewer’s concerns and that our revised manuscript is now suitable for publication in PLOS ONE.

**Reviewer 1**

We thank Reviewer #1 for the critical comments and useful suggestions, which have substantially helped us improve our manuscript. As indicated in our responses below, we have considered each reviewer comment and suggestion and have revised the manuscript accordingly. We hope that our responses and revisions are appropriate and that our revised manuscript is considered for publication.

1. **“Reviewer’s comment”**

Abstract: The introduction in the abstract makes the reader think that only steps from walking around the house and from fidgeting will be taken into consideration for this study. Since this is not the case, this should be removed.

**“Author’s response”**

We agreed with reviewer’s comment. We have removed this sentence and added the sentence in the Abstract section, as follows:

P 2, line 15- 16

There is increasing evidence that physical activity is associated with sleep quality.

2. **“Reviewer’s comment”**

Introduction: Page 4, line 63- the authors mention that only one study has assessed the relationship between MVPA and sleep parameters, however, there have been other studies conducted on the subject matter -Gabriel, Kelley Pettee, et al. "Bidirectional associations of accelerometer-determined sedentary behavior and physical activity with reported time in bed: Women's Health Study." Sleep health 3.1 (2017): 49-55.

**“Author’s response”**

We agreed with reviewer’s comment. We have revised the relevant sentences in the Methods section, as follows:

P 4, line 60- 63

To the best of our knowledge, a few studies have reported the relationship between objectively measured moderate-to-vigorous- intensity physical activities and sleep parameters in community-dwelling older adults [15, 16].

3. **“Reviewer’s comment”**

The authors state that they will focus on daily walking steps since it is a measure of low-intensity activity-is there a citation for this? How are the authors able to discern whether steps taken belonged in the light or moderate-to-vigorous intensity category? The authors may consider not talking about intensity since it is not reporting OR they may consider reporting time spent in light, moderate and vigorous intensity.

**“Author’s response”**

Several previous studies reported that walking may be categorized into light-intensity physical activity, as follows:

“Whether the association includes low-intensity activity such as regular walking is not known.” Abbott RD, et al. JAMA. 2004;292:1447-1453.

“Women may　spend more time doing low and lifestyle intensity activities,　such as walking, household chores, and gardening.”　Lohne-Seiler et al. BMC Public Health 2014, 14:284.

“Recent studies have　shown that light-intensity PA (LPA; e.g., housework, gardening,　and casual walking) and sedentary behavior (SB;e.g., television viewing, computer use, workplace sitting,　and sitting in automobile) are also related to health of older　adults.”　Yasunaga A, et al. Health and Quality of Life Outcomes. 2018;16:240.

“These activities can include non-exercise leisure-time and life-style activities (e.g.　walking, gardening, etc.) and instrumental activities of daily living (IADLs) (e.g. shopping, housework, etc.), which are typically in the low-intensity range. Research using self-report measures of walking activity indicates that these non-exercise physical activities may be associated with cognitive health benefits.” Varma VR, et al. Hippocampus. 2015; 25: 605–615.

Therefore, we classified walking steps into light-intensity activity. However, we agreed with reviewer’s comment that walking steps include the light or moderate-to-vigorous intensity activity. Moreover, in the present study, we have not investigated the intensity of physical activity. Therefore, we have revised the relevant sentences in the Introduction and Discussion sections, as follows:

P 4, line 64- 68

In this study, we focused on daily walking steps because engaging in exercise programs is typically difficult for older people because of their physical limitations or health conditions. Walking is a convenient and safe activity for all age groups and accounts for most of the energy expenditure among older people.

P 11, line 208- 209

In contrast, we measured daily walking in a large number of older adults.

4. **“Reviewer’s comment”**

Methods:

Page 5, Line 83- how did the authors ensure “physical and psychological health”? What if they had a chronic disease?

**“Author’s response”**

We agreed with reviewer’s comment. We have added the relevant sentences in the Discussion section, as follows:

P 11, line 229- P 12, line 231

Although all participants were decided to be physically and psychologically healthy by physical examination medical interview at baseline, the participants with chronic disease could not be excluded completely from participating in the current study.

5. **“Reviewer’s comment”**

Discussion: Page 10, line 191, citation missing for “several studies” which ones?

**“Author’s response”**

We agreed with reviewer’s comment. We have revised the relevant sentences in the Discussion section, as follows:

P 10, line 192

Several studies have examined the association between physical function or activity and sleep parameters in older adults [10, 11, 14, 15, 22–26].

6. **“Reviewer’s comment”**

There are several grammatical errors throughout the manuscript.

Keywords: Community is misspelled

Page 3, line 54- Remove “almost”

Page 9, line 179, nor should be “not”Discussion:

**“Author’s response”**

We agreed with reviewer’s comment. We have corrected grammatical errors throughout the manuscript.
